# Knowledge, awareness, and attitude towards infection prevention and management among surgeons: identifying the surgeon champion

**DOI:** 10.1186/s13017-018-0198-x

**Published:** 2018-08-17

**Authors:** Massimo Sartelli, Yoram Kluger, Luca Ansaloni, Federico Coccolini, Gian Luca Baiocchi, Timothy C. Hardcastle, Ernest E. Moore, Addison K. May, Kamal M. F. Itani, Donald E. Fry, Marja A. Boermeester, Xavier Guirao, Lena Napolitano, Robert G. Sawyer, Kemal Rasa, Fikri M. Abu-Zidan, Abdulrashid K. Adesunkanmi, Boyko Atanasov, Goran Augustin, Miklosh Bala, Miguel A. Cainzos, Alain Chichom-Mefire, Francesco Cortese, Dimitris Damaskos, Samir Delibegovic, Zaza Demetrashvili, Belinda De Simone, Therese M. Duane, Wagih Ghnnam, George Gkiokas, Carlos A. Gomes, Andreas Hecker, Aleksandar Karamarkovic, Jakub Kenig, Vladimir Khokha, Victor Kong, Arda Isik, Ari Leppäniemi, Andrey Litvin, Eftychios Lostoridis, Gustavo M. Machain, Sanjay Marwah, Michael McFarlane, Cristian Mesina, Ionut Negoi, Iyiade Olaoye, Tadeja Pintar, Guntars Pupelis, Miran Rems, Ines Rubio-Perez, Boris Sakakushev, Helmut Segovia-Lohse, Boonying Siribumrungwong, Peep Talving, Jan Ulrych, András G. Vereczkei, Francesco M. Labricciosa, Fausto Catena

**Affiliations:** 1 Department of Surgery, Macerata Hospital, Via Santa Lucia, 62100 Macerata, Italy; 2 Department of General Surgery, Division of Surgery, Rambam Health Care Campus, Haifa, Israel; 3 General Surgery Department, “M. Bufalini” Cesena Hospital, Cesena, Italy; 4 Department of Clinical and Experimental Sciences, University of Brescia, Brescia, Italy; 5 Department of Surgery, Trauma Service, Inkosi Albert Luthuli Central Hospital, Nelson R Mandela School of Clinical Medicine, Durban, South Africa; 6 Department of Surgery, Denver Health Medical Center, University of Colorado, Denver, CO USA; 7 Division of Trauma and Surgical Critical Care, Vanderbilt University Medical Center, Nashville, TN USA; 8 Department of Surgery, VA Boston Health Care System, Boston University, Harvard Medical School, Boston, MA USA; 9 Department of Surgery, Northwestern University Feinberg School of Medicine, Chicago, IL USA; 10 University of New Mexico School of Medicine, Albuquerque, NM USA; 11 Department of Surgery, Academic Medical Center, Amsterdam, Netherlands; 12 Unit of Endocrine, Head, and Neck Surgery and Unit of Surgical Infections Support, Department of General Surgery, ParcTaulí, Hospital University, Sabadell, Spain; 13 Department of Surgery, University of Michigan, Ann Arbor, MI USA; 14 Department of Surgery, Western Michigan University School of Medicine, Kalamazoo, MI USA; 15 Department of Surgery, Anadolu Medical Center, Kocaali, Turkey; 16 Department of Surgery, College of Medicine and Health Sciences, UAE University, Al-Ain, United Arab Emirates; 17 Department of Surgery, College of Health Sciences, Obafemi Awolowo University, Ile-Ife, Nigeria; 18 Department of General Surgery, Medical University of Plovdiv, UMHAT Eurohospital, Plovdiv, Bulgaria; 19 Department of Surgery, University Hospital Centre Zagreb, Zagreb, Croatia; 20 Trauma and Acute Care Surgery Unit, Hadassah Hebrew University Medical Center, Jerusalem, Israel; 21 Department of Surgery, Hospital Clínico Universitario, Santiago de Compostela, Spain; 22 Department of Surgery and Obstetrics/Gynaecology, Regional Hospital, Limbe, Cameroon; 23 Emergency Surgery Unit, San Filippo Neri Hospital, Rome, Italy; 24 Department of Surgery, Royal Infirmary of Edinburgh, Edinburgh, UK; 25 Department of Surgery, University Clinical Center of Tuzla, Tuzla, Bosnia and Herzegovina; 26 Department General Surgery, Kipshidze Central University Hospital, Tbilisi, Georgia; 27 Department of Digestive Surgery, Cannes Hospital, Cannes, France; 28 Department of Surgery, John Peter Smith Health Network, Fort Worth, TX USA; 29 Department of General Surgery, Mansoura Faculty of Medicine, Mansoura University, Mansoura, Egypt; 30 Second Department of Surgery, Aretaieion University Hospital, National and Kapodistrian University of Athens, Athens, Greece; 31 Faculdade de Ciências Médicas e da Saúde de Juiz de Fora, Department of Surgery, Hospital Universitário Terezinha de Jesus, Juiz de Fora, Brazil; 32 Department of General and Thoracic Surgery, University Hospital Giessen, Giessen, Germany; 33 Clinic for Surgery, University Clinical Center “Zvezdara”, Belgrade, Serbia; 34 Third Department of General Surgery, Jagiellonian University Medical College, Krakow, Poland; 35 Department of Emergency Surgery, City Hospital, Mozyr, Belarus; 36 Department of Surgery, Edendale Hospital, Pietermaritzburg, South Africa; 37 General Surgery Department, Magee Womens Hospital, UPMC, Pittsburgh, USA; 38 Abdominal Center, University Hospital Meilahti, Helsinki, Finland; 39 Surgical Disciplines, Immanuel Kant Baltic Federal University/Regional Clinical Hospital, Kaliningrad, Russian Federation; 40 1st Department of Surgery, Kavala General Hospital, Kavala, Greece; 41 Department of Surgery, II Cátedra de Clínica Quirúrgica, Universidad Nacional de Asunción, San Lorenzo, Paraguay; 42 Department of Surgery, Post-Graduate Institute of Medical Sciences, Rohtak, India; 43 Department of Surgery, Radiology, University Hospital of the West Indies, Kingston, Jamaica; 44 Second Surgical Clinic, Emergency Hospital of Craiova, Craiova, Romania; 45 Department of Surgery, Emergency Hospital of Bucharest, Bucharest, Romania; 46 Department of Surgery, University of Ilorin Teaching Hospital, Ilorin, Nigeria; 47 Department of Surgery, UMC Ljubljana, Ljubljana, Slovenia; 48 Department of General and Emergency Surgery, Riga East University Hospital “Gailezers”, Riga, Latvia; 49 Department of General Surgery, Jesenice General Hospital, Jesenice, Slovenia; 50 Colorectal Surgery Unit, General Surgery Department, La Paz University Hospital, Madrid, Spain; 51 General Surgery Department, Medical University, University Hospital St George, Plovdiv, Bulgaria; 52 Faculty of Medicine, Department of Surgery, Thammasat University Hospital, Thammasat University, Amphoe Khlong Luang, Pathum Thani Thailand; 53 Department of Surgery, North Estonia Medical Center, Tallinn, Estonia; 54 First Department of Surgery-Department of Abdominal, Thoracic Surgery and Traumatology, General University Hospital, Prague, Czech Republic; 55 Department of Surgery, Medical School University of Pécs, Pécs, Hungary; 56 Global Alliance for Infections in Surgery, Porto, Portugal; 57 Department of Emergency Surgery, Maggiore Hospital, Parma, Italy

**Keywords:** Surgeon, Infection, Prevention, Antibiotic therapy

## Abstract

Despite evidence supporting the effectiveness of best practices of infection prevention and management, many surgeons worldwide fail to implement them. Evidence-based practices tend to be underused in routine practice. Surgeons with knowledge in surgical infections should provide feedback to prescribers and integrate best practices among surgeons and implement changes within their team. Identifying a local opinion leader to serve as a champion within the surgical department may be important. The “surgeon champion” can integrate best clinical practices of infection prevention and management, drive behavior change in their colleagues, and interact with both infection control teams in promoting antimicrobial stewardship.

## Background

In 2017, a global declaration for appropriate use of antimicrobials across the surgical pathway was shared by over 230 experts from 83 different countries [1]. Within this declaration, the authors highlighted the effects of antibiotic exposure, misuse, and overuse on antibiotic resistance and outlined the fundamental principles of appropriate antibiotic prophylaxis and therapy in surgery.

In that declaration, efforts to prevent healthcare-associated infections (HAIs) were not specifically highlighted [2]. HAIs including surgical site infection, ventilator-associated pneumonia, central line-associated bloodstream infection, and catheter-associated urinary tract infection are the most common nosocomial infections. Healthcare-associated infections continue to be of significant importance in surgical patients.

Surgical site infections (SSIs) are a major clinical problem in terms of morbidity, mortality, length of hospital stay, and overall direct and indirect costs globally [3, 4]. However, knowledge, attitudes, and awareness of infection prevention and control measures vary significantly among surgeons. Most significantly, a gap seems to exist between best evidence and clinical practice with regard to SSIs prevention.

The World Health Organization (WHO) [5], the Centers for Disease Control and Prevention (CDC) [6], and a combined effort by the American College of Surgeons/Surgical Infection Society [7, 8] have recently published guidelines for the prevention of SSIs. Despite clear evidence and guidelines to direct SSIs prevention strategies, compliance is unacceptably poor [9].

Preoperative antibiotic prophylaxis (PAP) is a cornerstone of SSIs prevention. The use of PAP contributes considerably to the total amount of antibiotics used in hospitals worldwide. PAP has been shown to be an effective measure for the prevention of SSIs, but its use should be limited to specific, well-accepted indications to avoid cost, toxicity, and antimicrobial resistance, and should never substitute for the good medical practice of infection prevention and control. Guidelines stress the importance of cessation of antibiotic prophylaxis immediately after surgery and refrain from the extension of prophylaxis outside the operating theater [5]. High rates of inappropriate use of prophylactic antibiotics in surgery continue to be reported in the literature [9]. In Appendix 1, seven strategies for correct antibiotic prophylaxis are illustrated.

Antibiotic therapy is an additional key component of daily surgical practice. Antibiotics are life-saving when treating bacterial infections but are often used inappropriately, specifically when not indicated. Antibiotic therapy plays an integral role in the management of surgical infections, especially in critically ill patients who require immediate empiric antibiotic therapy. Poor antibiotic coverage and inappropriate regimens are the variables most strongly associated with unfavorable outcomes [10, 11]. The timing, regimen, dose, route of administration, and duration of antibiotic therapy should be always optimized. In most patients with surgical infections, antibiotic therapy aims to treat any residual infection after adequate source control. In these patients, prolonging antibiotic treatment, which can lead to antibiotic resistance, does not prevent the persistence or recurrence of the infection. When source control is obtained, the duration of antibiotic therapy should be shortened as much as possible, unless there are special circumstances that require prolonging antimicrobial therapy, such as signs of an ongoing infection. Patients who have systemic signs of sepsis beyond 5 to 7 days of treatment warrant diagnostic investigation to determine an ongoing uncontrolled source of infection needing intervention [8]. In Appendix 2, ten strategies for correct antibiotic therapy are illustrated.

## The surgeon as a champion in preventing and treating infections

Surgeons prescribing antibiotics have two opposing responsibilities. They have to (1) offer optimal therapy for the individual patient under their care and (2) restrain from overprescribing antibiotics to preserve their efficacy and minimize the rate of emergence of antimicrobial resistance, while also preventing collateral damage from antibiotics (such as *Clostridium difficile* infections) [12].

Because most surgeons have already established their attitudes and behaviors with regard to antibiotic usage, it is difficult to change their deeply established views and practice patterns. A range of factors such as diagnostic uncertainty, fear of clinical failure, time pressure, or organizational issues can influence prescribing decisions. However, due to cognitive dissonance (recognizing that an action is necessary but not implementing it), it is very challenging to change the prescribing behavior [1].

Surgeons should be aware of their role and responsibility for maintaining the effectiveness of current and future antibiotics. Although most of them are aware of the problem of antimicrobial resistance (AMR), many still underestimate this problem in their own hospital and in their patients. Inappropriate use of antibiotics, as well as poor prevention and control of infections such as hand hygiene, is contributing to the development of AMR. Surgeons are at the forefront in managing patients with infections and should take a proactive role in ensuring both effective antibiotic therapy and the avoidance of inappropriate and unnecessary antibiotic exposure [12].

Successful and cost-effective strategies to reduce AMR should involve a multi-faceted approach aimed at optimizing antibiotic use, strengthening surveillance and infection prevention and control, and improving clinician education regarding the appropriate use of antibiotics.

A growing body of evidence demonstrates that hospital-based programs dedicated to improving antibiotic use can both optimize the treatment of infections and reduce the adverse events associated with antibiotic use [1]. “Antibiotic stewardship programs” (ASPs) may significantly reduce the incidence of antimicrobial resistance and *Clostridium difficile* infections in hospitalized patients [1]. Every hospital worldwide should utilize the existing resources to create effective ASPs with a multi-disciplinary team. However, the best strategies to establish an ASPs are not well defined.

ASPs should be linked to infection control and prevention programs in order to improve antibiotic prescribing practices and to prevent infections.

Dedicated efforts, such as the establishment of locally adopted multi-disciplinary, evidence-based protocols and guidelines; unit specific antibiotic sensitivity data; and compliance monitoring, are required to maximize the performance.

To investigate the effects of the antibiotic stewardship program on prevention and control of SSIs during clean surgery, Liu et al. [13] compared the effect before and after an antibiotic stewardship program intervention. From January 1, 2010, to December 31, 2016, 41,426 patients underwent clean surgeries in a grade III, class A hospital. The rate of prophylactic antibiotic use in the 41,426 clean surgeries was reduced from 82.9 to 28.0% after the interventions. The rate of antibiotic agents administered within 120 min of the first incision increased from 20.8 to 85.1%. The rate at which prophylactic antimicrobial agents were discontinued in the first 24 h after surgery increased from 22.1 to 60.4%. Appropriate antibiotic selection increased from 37.0 to 93.6%. Prophylactic antibiotic re-dosing increased from 3.8 to 64.8%. The SSI rate decreased from 0.7 to 0.5% (*p* < 0.05). The pathogen detection rate increased from 16.7 up to 41.8% after intervention. The intensity of antibiotic consumption reduced from 74.9 defined daily doses (DDDs) per 100 bed-days to 34.2 DDDs per 100 bed-days after the interventions.

Van Kasteren et al. [14] in a prospective multi-site study of elective procedures in 13 Dutch hospitals evaluated the quality of prophylaxis auditing before and after an intervention consisting of performance feedback and implementation of national clinical practice guidelines. Antimicrobial use decreased from 121 to 79 DDD/100 procedures, and costs reduced by 25% per procedure. After the intervention, the antibiotic choice was inappropriate in only 37.5% of the cases instead of in 93.5% expected cases prior to the intervention. Prolonged prophylaxis was observed in 31.4% instead of 46.8% expected cases and inappropriate timing in 39.4% instead of the expected 51.8%. Time series analysis showed that all improvements were statistically significant (*p* < 0.01). The overall SSI rates before and after intervention were 5.4% (95% CI 4.3–6.5) and 4.6% (95% CI 3.6–5.4), respectively.

Raising awareness is a crucial factor in changing behaviors. Efforts to improve educational programs are thus required, and this should preferably be complemented by active interventions such as prospective audits and feedback to clinicians to stimulate further changes. It would be advisable to introduce specific courses and training about antibiotics in the core curriculum of medical students, with emphasis on the rational prescription of antibiotics that emphasizes more the behavior of medical students towards antibiotics use rather than the advance of knowledge alone [15].

The best means of improving ASPs worldwide should involve collaboration among various specialties within a healthcare institution including prescribing clinicians. ASPs have many contributors, steps, and actions specifically related to the prevention and management of infection. The multidisciplinary approach reinforces the concept that all professionals bring with them their particular expertise and are responsible for their respective contributions. In this context, the direct involvement of surgeons can be highly effective.

Surgeons with adequate knowledge in surgical infection who are involved in an ASPs may provide feedback and integrate the best practices of infection prevention and treatment among surgeons.

Very few studies have focused on the relationship between ASPs and surgeons. In 2011, Dortch et al. [16] reported the results of a surgeon-led infection reduction and antibiotic stewardship program in two separate ICUs, one trauma and one surgical. Components of the program included unit-specific empiric and therapeutic antibiotic protocols for healthcare-acquired infections, prophylaxis guidelines, and numerous infection reduction strategies. They demonstrated a marked reduction in infectious complications, multidrug-resistant pathogens, and broad-spectrum antibiotic use.

In 2015, Çakmakçi [17] suggested that the engagement of surgeons in ASPs may be crucial to their success. However, Duane et al. [18] showed poor compliance of surgical services with ASP recommendations.

A retrospective study published in 2016 showed [19] that the implementation of an educational and surveillance-based ASP achieves a significant improvement in all antibiotic agent prescriptions and a reduction in antibiotics consumption. In a surgical unit performing mainly elective major abdominal surgery and emergency surgery, both a local protocol of surgical prophylaxis and a set of guidelines for management of intra-abdominal infections (IAIs) were introduced [20]. Moreover, a unit-specific control of antimicrobial agents used and surveillance of antimicrobial resistance were implemented. Comparing the pre-intervention and post-intervention periods, the mean total monthly antimicrobial use decreased significantly after the intervention. The model was based on the concept of the “surgeon champion.” The “champion” was a surgeon who on a day-to-day basis worked within the surgical unit, promoting and maintaining a culture in which both infection prevention and management were given high priority. Such a champion model has been previously applied to surgical safety implementations in general, such as surgical checklists, and plays a key role in successful quality improvement at the hospital level [21].

Identifying a local opinion leader to serve as a champion may be important because the “surgeon champion” may integrate best clinical practices and drive their colleagues into changing behaviors while maintaining a tight collaboration with antimicrobial stewardship and infection control teams. The “champion” should possess a wide knowledge of surgical microbiology, as well as considerable expertise in the antibiotic management of surgical infections. Furthermore, the “champion” should increase the awareness of surgical infections in young staff surgeons and surgical residents.

We think that the concept of the “surgeon champion” can be a crucial way to improve the infection prevention and antibiotic prescribing practices across the surgical practice pathway.

## Conclusions

Surgeons must be aware that appropriate antibiotic utilization is an integral part of any stewardship program and necessary to maximize clinical cure and minimize the emergence of antimicrobial resistance.

Antimicrobial restriction is not more effective than the persuasive strategy in achieving the goal of controlling antimicrobial use in the long term [22]. Moreover, in many settings, there may be inadequate personnel for a restrictive approach, and restriction strategies fail to consider the appropriateness of use of non-restricted antibiotics, which makes up the vast majority of antibiotics used in the hospital [23]. The impact on surgeon autonomy with antimicrobial restriction may also create barriers to collaboration with members of the ASP resulting in less communication about stewardship. Therefore, the emphasis needs to be on the incorporation of a surgeon champion in the ASPs.

Surgeons with good knowledge of surgical infections involved in ASPs may audit antibiotic prescriptions, provide feedback to the prescribers, and integrate the best practice of infection prevention and management among surgeons. We think that the concept of the “surgeon champion” is a crucial way to improve infection prevention and antibiotic prescribing practices across the surgical practice pathway.

## Appendix 1

Strategies for a correct antibiotic prophylaxis [1]:

1. Antibiotics alone are unable to prevent surgical site infections. Strategies to prevent surgical site infections should always include attention to infection and prevention control strategies including correct and compliant hand hygiene practices
2. Antibiotic prophylaxis should be administered for operative procedures that have a high rate of postoperative surgical site infection, or when foreign materials are implanted.
3. Antibiotics given as prophylaxis should be effective against the aerobic and anaerobic pathogens most likely to contaminate the surgical site, i.e., Gram-positive skin commensals or normal flora colonizing the incised mucosae.
4. Antibiotic prophylaxis should be administered within 120 min prior to the incision. However, administration of the first dose of antibiotics beginning within 30–60 min before surgical incision is recommended for most antibiotics (e.g., cefazolin), to ensure adequate serum and tissue concentrations during the period of potential contamination. Obese patients require higher doses of antibiotic.
5. A single dose is generally sufficient. Additional antibiotic doses should be administered intraoperatively for procedures > 2–4 h (typically where duration exceeds two half-lives of the antibiotic) or with associated significant blood loss (> 1.5 L).
6. There is no evidence to support the use of postoperative antibiotic prophylaxis.
7. Each institution is encouraged to develop guidelines for the proper surgical prophylaxis.

## Appendix 2

Strategies for a correct antibiotic therapy [1]:

1. The source of infection should always be identified and controlled as soon as possible.
2. Antibiotic empiric therapy should be initiated after a treatable surgical infection has been recognized, since microbiological data (culture and susceptibility results) may not be available for up to 48–72 h to guide the targeted therapy.
3. In critically ill patients, empiric broad-spectrum therapy to cover all likely pathogens should be initiated as soon as possible after a surgical infection has been recognized. Empiric antimicrobial therapy should be narrowed once the culture and susceptibility results are available and/or adequate clinical improvement is noted. In patients less severely ill, it may be pertinent to await culture results.
4. Empirical therapy should be chosen on the basis of local epidemiology, individual patient risk factors for multidrug-resistant bacteria, clinical severity, and infection source.
5. Specimens for microbiological evaluation from the site of infection are always recommended for patients with hospital-acquired or with community-acquired infections at risk for resistant pathogens (e.g., previous antimicrobial therapy, prior infection or colonization with multidrug-resistant pathogens) and in critically ill patients. Blood cultures should be performed before the administration of antibiotics in critically ill patients.
6. Antibiotics dose should be optimized to ensure that pharmacodynamic-pharmacokinetic targets are achieved
7. The appropriateness and need for antimicrobial treatment should be reassessed daily.
8. Once the source control is established, short courses of antibiotic therapy are as effective as longer courses regardless of the signs of inflammation according to the guidelines.
9. Failure of antibiotic therapy in patients having continued evidence of active infection may require a re-operation for a second source control intervention.
10. Biomarkers such as procalcitonin may be useful to guide the duration and/or cessation of antibiotic therapy in critically ill patients.

## Abbreviations

AMR: Antimicrobial resistance
ASP: Antimicrobial stewardship program
HAIs: Healthcare-associated infections
PAP: Preoperative antibiotic prophylaxis
SSI: Surgical site infection
